# Liver Resection after Disease Control with Abemaciclib for Solitary Liver Metastasis from Breast Cancer: A Case Report

**DOI:** 10.70352/scrj.cr.26-0141

**Published:** 2026-06-05

**Authors:** Yuki Kurokawa, Hirofumi Terakawa, Ryosuke Mohri, Miki Hirata, Ryosuke Gabata, Shintaro Yagi, Noriyuki Inaki

**Affiliations:** 1 Department of Gastrointestinal Surgery/Breast Surgery, Kanazawa University Hospital, Kanazawa, Ishikawa, Japan; 2 Department of Hepato-Biliary-Pancreatic Surgery and Transplantation/Pediatric Surgery, Kanazawa University Hospital, Kanazawa, Ishikawa, Japan

**Keywords:** breast neoplasms, liver neoplasms, secondary, abemaciclib, cyclin-dependent kinase 4/6 inhibitors, liver resection

## Abstract

**INTRODUCTION:**

Liver metastases from breast cancer are associated with a poorer prognosis than bone or soft tissue metastases. In hormone receptor (HR)–positive, human epidermal growth factor receptor 2 (HER2)–negative recurrent or metastatic breast cancer, endocrine therapy combined with cyclin-dependent kinase 4/6 (CDK4/6) inhibitors has been established as the standard of care. Abemaciclib has demonstrated favorable efficacy in phase III clinical trials and is considered an important option for long-term disease control. With recent advances in systemic therapy, an increasing number of patients achieve sustained disease control; however, prolonged treatment may lead to impaired QOL, financial burden, and the need for long-term therapy. Consequently, the role of local treatment in carefully selected patients with well-controlled disease has increasingly been discussed. Here, we report a case of breast cancer with postoperative recurrent solitary liver metastasis in which disease control was achieved with endocrine therapy plus abemaciclib, followed by liver resection, resulting in long-term recurrence-free survival.

**CASE PRESENTATION:**

A 45-year-old woman with HR–positive, HER2–negative breast cancer underwent breast-conserving surgery followed by adjuvant chemotherapy, radiotherapy, and endocrine therapy. Five years after surgery, follow-up CT revealed a solitary liver lesion. Percutaneous biopsy demonstrated adenocarcinoma consistent with a solitary liver metastasis from breast cancer. Combination therapy with a luteinizing hormone–releasing hormone agonist, fulvestrant, and abemaciclib was initiated. Although dose reduction of abemaciclib was required because of grade 3 neutropenia, stable disease was maintained for approximately 1 year. Given the sustained disease control, absence of extrahepatic metastasis, and the patient’s strong preference, laparoscopic liver resection was performed after obtaining informed consent. Pathological examination revealed adenocarcinoma consistent with metastatic breast cancer, with negative surgical margins. Postoperatively, endocrine therapy alone was continued, and the patient has remained recurrence-free for 3 years after liver resection.

**CONCLUSIONS:**

This case represents a rare report of surgical resection for breast cancer liver metastasis after CDK4/6 inhibitor–based therapy. It suggests that local treatment may be effective even in cases with suspected endocrine-resistant disease and provides practical insight into patient selection and treatment strategies, as the case fulfilled previously reported criteria for local therapy.

## Abbreviations

AJCC
: American Joint Committee on Cancer
CDK4/6
: cyclin-dependent kinase 4/6
CTCAE
: Common Terminology Criteria for Adverse Events
ER
: estrogen receptor
FDG-PET/CT
: fluorodeoxyglucose PET/CT
HER2
: human epidermal growth factor receptor 2
HR
: hormone receptor
LH-RH
: luteinizing hormone–releasing hormone
PgR
: progesterone receptor
RECIST
: Response Evaluation Criteria in Solid Tumors
RFA
: radiofrequency ablation

## INTRODUCTION

Liver metastases from breast cancer are associated with a poorer prognosis compared with bone or soft tissue metastases.^1)^ In patients with HR–positive and HER2–negative recurrent or metastatic breast cancer, endocrine therapy combined with CDK4/6 inhibitors has been established as the current standard of care.^2)^ CDK4/6 inhibitors are a relatively recent class of agents, first introduced into clinical practice following the approval of palbociclib in the United States in 2015.^3)^ Among these agents, abemaciclib has demonstrated favorable clinical outcomes in phase III trials and is considered an important option for achieving long-term disease control.^4)^

With advances in systemic therapies including CDK4/6 inhibitors, an increasing number of patients achieve prolonged disease stabilization. However, long-term systemic therapy is associated with several challenges, including deterioration in QOL,^5)^ financial burden,^6)^ frequent hospital visits, reduced fertility,^7)^ and the need for long-term or potentially indefinite continuation of treatment. Against this background, the role of local treatment has increasingly been discussed in patients whose disease is well controlled by systemic therapy.

At present, surgical resection for liver metastases from breast cancer is not generally recommended. Nevertheless, recent studies have suggested that, in carefully selected patients, liver resection may be associated with prolonged survival.^8–11)^ Here, we report a case of postoperative recurrent solitary liver metastasis from breast cancer in which disease control was achieved with endocrine therapy combined with abemaciclib, followed by liver resection, resulting in long-term recurrence-free survival.

## CASE PRESENTATION

**Fig. 1:**
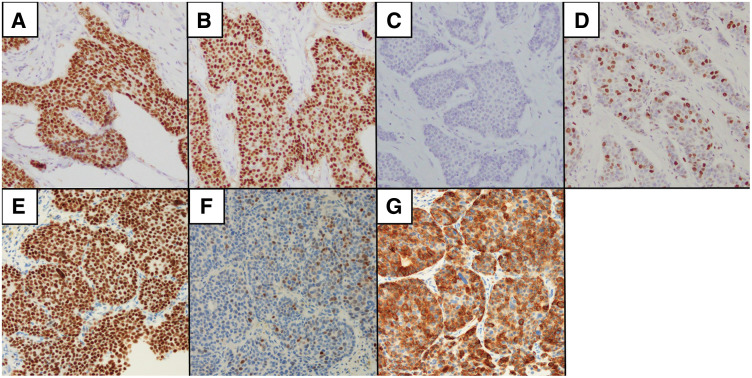
Immunohistochemical findings of the primary breast tumor and liver metastasis. (**A**–**D**) Primary tumor: ER (>90%), PgR (>90%), HER2-IHC 0, and Ki-67 index (18%). (**E**–**G**) Liver metastasis: ER (>90%), PgR (5%–10%), and CK7 (positive). HER2-IHC 0 and Ki-67 (<1%) of the metastatic lesion were assessed, but corresponding images were not available. CK7, cytokeratin 7; ER, estrogen receptor; HER2, human epidermal growth factor receptor 2; IHC, immunohistochemistry; PgR, progesterone receptor

A 45-year-old woman with no significant past medical history was diagnosed with right-sided breast cancer. The clinical stage according to the 8th edition of the AJCC staging system was cT2N0M0, stage IIA. The tumor subtype was HR-positive/HER2-negative, with immunohistochemical findings showing ER expression >90%, PgR expression >90%, a HER2 immunohistochemistry score of 0, and a Ki-67 index of 18% (**Fig. 1A**–**1D**).

The patient underwent breast-conserving surgery with sentinel lymph node biopsy. Postoperative pathological staging was pT2N1M0, stage IIB. One of 5 retrieved sentinel lymph nodes showed metastasis and axillary lymph node dissection was omitted. Adjuvant chemotherapy consisting of 4 cycles of docetaxel plus cyclophosphamide was administered, followed by radiotherapy to the conserved breast and axilla with a total dose of 60 Gy. Adjuvant endocrine therapy with tamoxifen was subsequently initiated.

**Fig. 2:**
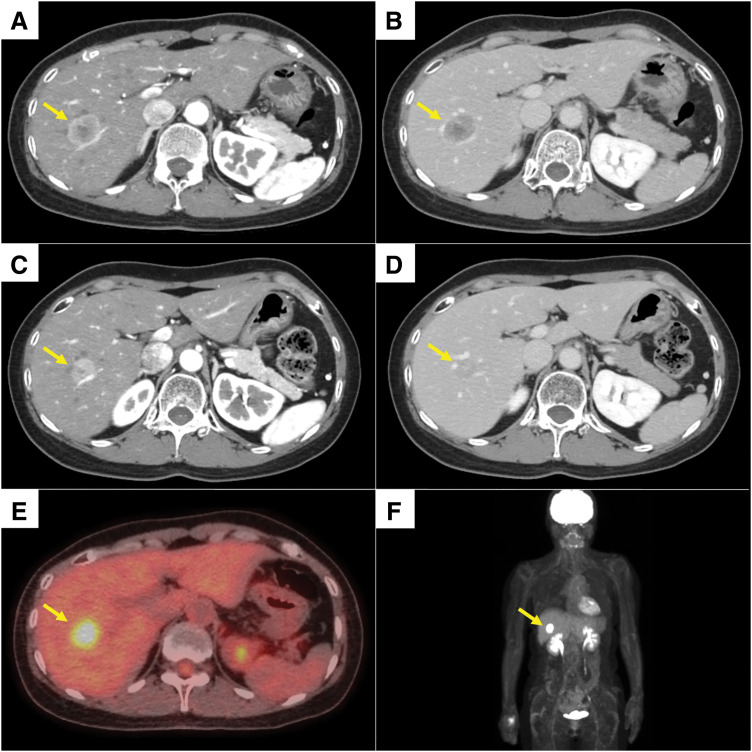
Contrast-enhanced CT and FDG-PET/CT images of the liver metastasis. The yellow arrows indicate the liver metastasis. (**A**) Arterial phase image at recurrence demonstrating a hyperenhancing lesion in segments 5 and 6 of the liver. (**B**) Delayed phase image showing relative hypoattenuation with peripheral enhancement. (**C**) Arterial phase image obtained after approximately 1 year of systemic therapy, demonstrating tumor shrinkage and reduced enhancement. (**D**) Delayed phase image after systemic therapy showing decreased lesion conspicuity compared with pretreatment imaging. (**E**) FDG-PET/CT image obtained after approximately 1 year of systemic therapy, demonstrating FDG uptake corresponding to the liver lesion (SUVmax: early 4.5, delayed 5.5). (**F**) MIP image showing no evidence of extrahepatic metastases; mild uptake in the right palm was considered physiological. At recurrence, the longest diameter of the liver metastasis was 28.5 mm. After approximately 1 year of systemic therapy, the lesion decreased in size to a longest diameter of 21.8 mm, corresponding to a tumor size reduction of 23.5% and classified as stable disease according to RECIST version 1.1. In both the recurrent and post-treatment (preoperative) images, the lesion demonstrated arterial phase hyperenhancement followed by relative washout in the delayed phase. FDG, fluorodeoxyglucose; MIP, maximum intensity projection; RECIST, Response Evaluation Criteria in Solid Tumors; SUVmax, maximum standardized uptake value

Five years after surgery, contrast-enhanced CT performed during routine follow-up revealed a solitary mass measuring 28.5 mm in segments 5 and 6 of the liver (**Fig. 2A** and **2B**). Percutaneous needle biopsy demonstrated adenocarcinoma consistent with liver metastasis from breast cancer. Immunohistochemical analysis of the liver metastasis showed ER expression >90%, PgR expression of 5%–10%, an HER2 immunohistochemistry score of 0, and a Ki-67 index of <1%. Cytokeratin 7 was also positive (**Fig. 1E**–**1G**). For recurrent disease, combination therapy with an LH-RH agonist, fulvestrant, and abemaciclib was initiated. Within 6 months after treatment initiation, the patient developed grade 3 neutropenia according to the CTCAE version 5.0, leading to a 2-step dose reduction of abemaciclib from 300 to 100 mg/day. Thereafter, treatment was continued without significant adverse events, although persistent grade 2 fatigue was observed, and the liver lesion remained controlled for approximately 1 year, with stable disease confirmed according to the RECIST version 1.1 (**Fig. 2C** and **2D**). Follow-up FDG-PET/CT demonstrated no evidence of new metastatic lesions (**Fig. 2E** and **2F**). Serum tumor markers, including carcinoembryonic antigen, cancer antigen 15-3, and NCC-ST439, remained within normal ranges throughout the clinical course, and were measured at regular intervals (every 6 months after initial diagnosis and every 3 months after detection of liver metastasis).

**Fig. 3:**
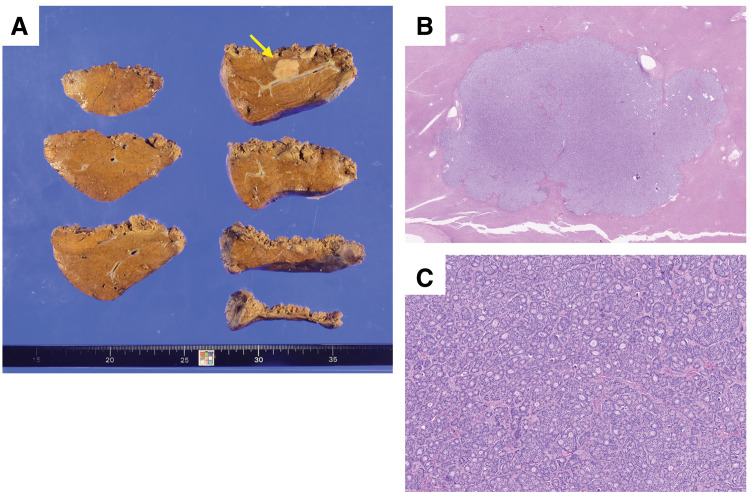
Pathological findings of the resected liver metastasis. (**A**) Macroscopic appearance of the resected specimen showing a whitish solid tumor measuring 18 × 17 mm (arrow). (**B**) H&E-stained section demonstrating the overall architecture of the tumor with negative surgical margins. Only minimal fibrosis was observed in the non-tumorous background liver tissue. (**C**) H&E-stained section at low magnification (×4) revealing proliferation of atypical epithelial cells with mildly pleomorphic, round-to-oval nuclei forming small tubular, fused tubular, and cribriform structures, consistent with adenocarcinoma. No evident necrosis or fibrosis was observed in the tumor. H&E, hematoxylin and eosin

Because of concerns regarding long-term oral therapy—primarily due to financial burden and persistent grade 2 fatigue—the patient strongly requested surgical resection of the metastatic lesion. Given the sustained disease control under systemic therapy and the absence of clinically evident extrahepatic disease, surgical resection was considered a reasonable local treatment option. After thorough explanation that this approach was not standard therapy and after obtaining informed consent, liver resection was planned as local therapy. Conversion to open surgery was anticipated if necessary, and informed consent was obtained preoperatively. Subsequently, laparoscopic posterior sectionectomy of the liver with cholecystectomy was performed. The postoperative course was uneventful, and the patient was discharged without complications. Histopathological examination revealed adenocarcinoma in the resected liver specimen, with findings consistent with metastatic breast cancer, and the surgical margins were negative (**Fig. 3A**–**3C**). No evident necrosis or fibrosis was observed in the tumor, and only minimal fibrosis was observed in the non-tumorous background liver tissue. After surgery, adjuvant endocrine therapy with letrozole alone was initiated, without significant adverse events. The patient has remained recurrence-free for 3 years following liver resection.

## DISCUSSION

The liver is the second most common site of distant metastasis in patients with breast cancer, following bone, and approximately half of patients with metastatic breast cancer develop liver metastases.^1)^ In most cases, liver metastases occur during disease progression and are frequently diagnosed as diffuse or multiple lesions and/or with concomitant metastases to other organs. In contrast, liver-limited metastasis at diagnosis is relatively rare, accounting for less than 5% of cases.^1)^ Historically, the prognosis of breast cancer with liver metastasis has been poor, with reported median survival times of 22–27 months in patients with liver-only metastases and 9–14 months in those with both liver and extrahepatic metastases treated with taxane-based systemic therapy.^12)^

Subtype-specific analyses have shown that patients with HR-positive/HER2-negative breast cancer and liver metastases have the second poorest prognosis after those with triple-negative disease, with reported median progression-free survival of approximately 7–8 months and overall survival of 22–25 months.^13)^ In contrast, patients with HER2-positive disease tend to have more favorable outcomes, particularly when HER2 expression is confirmed or newly identified by re-biopsy of metastatic lesions.^14)^

In recent years, endocrine therapy combined with CDK4/6 inhibitors has become the standard treatment for HR-positive/HER2-negative advanced or metastatic breast cancer.^2)^ In phase III clinical trials, abemaciclib demonstrated favorable outcomes, with a median progression-free survival of 29.0 months and a median overall survival of 66.8 months when used as first-line therapy.^4)^ Clinical benefit has also been observed in patients with visceral metastases, including liver involvement. However, these trials were not designed to specifically evaluate patients with liver metastases, and the optimal treatment strategy for this population remains unclear. In addition, adverse events such as diarrhea and fatigue, the psychological burden associated with long-term treatment, frequent hospital visits, and high medical costs represent important challenges in real-world practice.

Systemic therapy remains the cornerstone of treatment for breast cancer liver metastases, and liver resection is not currently recommended as standard care. Nevertheless, based on the concept of oligometastatic disease proposed by Hellman and Weichselbaum, a subset of patients with limited metastatic burden may achieve long-term disease control with local therapy.^15)^

Furthermore, systemic therapy not only reduces tumor burden but also exerts selective pressure on tumor clones.^16)^ As a result, disseminated aggressive clones may be suppressed, while residual disease may remain localized, potentially allowing local therapy to be more effective. In addition, recent retrospective studies have suggested that liver resection may be associated with prolonged survival in selected patients, defined as those with a solitary or limited number of liver metastases, good disease control under systemic therapy, no evidence of uncontrolled extrahepatic disease, technically feasible R0 resection, and adequate performance status to tolerate surgery.^8–11)^ A systematic review including 1686 patients from 43 studies reported a median overall survival of 36 months after liver resection, suggesting favorable outcomes in selected populations.^8)^ Subtype-based analyses have further indicated a potential survival benefit in patients with HER2-positive disease and in biologically aggressive HR-positive/HER2-negative tumors corresponding to the luminal B subtype.^11)^ Taken together, both tumor biological considerations and findings from previous clinical studies support the rationale for surgical resection in the present case, suggesting that this approach was reasonable.

However, these findings are derived from non-randomized retrospective studies and are therefore subject to selection bias and heterogeneity. A prospective RCT comparing systemic therapy alone with combined systemic and surgical treatment, the BRECLIM trial (NCT04079049), is currently ongoing.^17)^

RFA is a potential local treatment option for breast cancer liver metastases. However, when complete resection is feasible, surgical resection may provide more favorable long-term outcomes.^18)^ In this case, if RFA had been selected, the tumor location adjacent to the right hepatic vein may have reduced the efficacy of thermal ablation due to the heat sink effect,^19)^ and tumors near major vessels are associated with higher local recurrence rates.^20)^ Therefore, surgical resection was selected.

In the present case, the patient initially underwent breast-conserving surgery with sentinel lymph node biopsy, and axillary lymph node dissection was omitted in accordance with current guidelines based on the ACOSOG Z0011 trial.^21)^ Although PgR expression was decreased in the liver metastasis, HR and HER2 status are known to change between primary and metastatic lesions,^22)^ and cytokeratin 7 positivity further supported the diagnosis of metastatic breast cancer. At recurrence, systemic therapy with fulvestrant plus abemaciclib was selected based on evidence from the MONARCH 2 trial.^23)^ The patient had a solitary liver metastasis from HR-positive/HER2-negative breast cancer that remained stable for approximately 1 year under endocrine therapy combined with abemaciclib. The absence of extrahepatic disease and sustained tumor control with systemic therapy were consistent with previously reported criteria for considering liver resection. Furthermore, the relatively high Ki-67 index suggested a biologically aggressive tumor subtype in which liver resection has been associated with survival benefit in retrospective studies. The patient achieved recurrence-free survival for 3 years following liver resection. Notably, when calculated from the initiation of abemaciclib, the recurrence-free interval extended to 4 years, exceeding the median progression-free survival reported in clinical trials of abemaciclib. Postoperatively, disease control was maintained with endocrine therapy alone, potentially reducing treatment intensity, preserving QOL, and alleviating financial burden.

## CONCLUSIONS

This case represents a rare report of surgical resection for breast cancer liver metastasis after CDK4/6 inhibitor–based therapy. It suggests that local treatment may be effective even in cases with suspected endocrine-resistant disease and provides practical insight into patient selection and treatment strategies, as the case fulfilled previously reported criteria for local therapy.

## DECLARATIONS

### Availability of data and materials

The data supporting the findings of this study are not publicly available due to patient privacy concerns but are available from the corresponding author on reasonable request.
